# Evidencing the Impact of Web-Based Coproduction With Youth on Mental Health Research: Qualitative Findings From the MindKind Study

**DOI:** 10.2196/42963

**Published:** 2023-06-19

**Authors:** Blossom Fernandes, Lakshmi Neelakantan, Himani Shah, Sushmita Sumant, Pamela Y Collins, Jennifer Velloza, Emily Bampton, Swetha Ranganathan, Refiloe Sibisi, Toiba Bashir, Joshua Bowes, Esther Larisa David, Harsimar Kaur, Umairah Malik, Issy Shannon, Suvlaxmi Gurumayum, Anne-Marie Burn, Solveig K Sieberts, The MindKind Consortium, Mina Fazel

**Affiliations:** 1 Department of Psychiatry University of Oxford Oxford United Kingdom; 2 Centre for Mental Health, School of Population and Global Health, University of Melbourne Melbourne Australia; 3 Centre for Mental Health Law & Policy Indian Law Society Pune India; 4 Department of Psychiatry & Behavioral Sciences, University of Washington Seattle, WA United States; 5 Department of Global Health, University of Washington Seattle, WA United States; 6 Department of Epidemiology and Biostatistics, University of California San Francisco San Francisco, CA United States; 7 Higher Health, Higher Education and Training: Health, Wellness, and Development Centre Pretoria South Africa; 8 Mindkind Young People’s Advisory Group, Centre for Mental Health Law & Policy Indian Law Society Pune India; 9 Mindkind Young People’s Advisory Group, Department of Psychiatry University of Oxford Oxford United Kingdom; 10 Department of Psychiatry University of Cambridge Cambridge United Kingdom; 11 Sage Bionetworks Seattle, WA United States

**Keywords:** web-based youth coproduction, mental health, public involvement, young people, advisory groups

## Abstract

**Background:**

Public involvement in research is a growing phenomenon as well as a condition of research funding, and it is often referred to as coproduction. Coproduction involves stakeholder contributions at every stage of research, but different processes exist. However, the impact of coproduction on research is not well understood. Web-based young people’s advisory groups (YPAGs) were established as part of the MindKind study at 3 sites (India, South Africa, and the United Kingdom) to coproduce the wider research study. Each group site, led by a professional youth advisor, conducted all youth coproduction activities collaboratively with other research staff.

**Objective:**

This study aimed to evaluate the impact of youth coproduction in the MindKind study.

**Methods:**

To measure the impact of web-based youth coproduction on all stakeholders, the following methods were used: analysis of project documents, capturing the views of stakeholders using the *Most Significant Change* technique, and impact frameworks to assess the impact of youth coproduction on specific stakeholder outcomes. Data were analyzed in collaboration with researchers, advisors, and YPAG members to explore the impact of youth coproduction on research.

**Results:**

The impact was recorded on 5 levels. First, at the paradigmatic level, a novel method of conducting research allowed for a widely diverse group of YPAG representations, influencing study priorities, conceptualization, and design. Second, at the infrastructural level, the YPAG and youth advisors meaningfully contributed to the dissemination of materials; infrastructural constraints of undertaking coproduction were also identified. Third, at the organizational level, coproduction necessitated implementing new communication practices, such as a web-based shared platform. This meant that materials were easily accessible to the whole team and communication streams remained consistent. Fourth, at the group level, authentic relationships developed between the YPAG members, advisors, and the rest of the team, facilitated by regular web-based contact. Finally, at the individual level, participants reported enhanced insights into mental well-being and appreciation for the opportunity to engage in research.

**Conclusions:**

This study revealed several factors that shape the creation of web-based coproduction, with clear positive outcomes for advisors, YPAG members, researchers, and other project staff. However, several challenges of coproduced research were also encountered in multiple contexts and amid pressing timelines. For systematic reporting of the impact of youth coproduction, we propose that monitoring, evaluation, and learning systems be designed and implemented early.

## Introduction

### Background

Increasingly, participatory and collaborative research methods have been used to reduce the gap between evidence and implementation [1-3]. One way to do this is to involve target groups in research, using coproduction processes, particularly the involvement of youth, to reflect their needs in research [4,5]. Coproduction is “an approach in which researchers, practitioners and the public work together, sharing power and responsibility from the start to the end of the project, including the generation of knowledge” [6]. Coproduction aims to ensure that the knowledge generated is informed by the needs of the target group, is relevant to them, and has greater applicative value in their local setting [7]. This is somewhat different from what Baum et al [8] describe as participatory action research, where participants are involved in a reflective manner, by collecting and analyzing the data and then determining the outcome. Thus, the active involvement of knowledge users (eg, young people with mental health difficulties) in research, who are experts in their experience, aims to enhance the quality and utility of research.

### Coproduction in Mental Health Research With Youth

Different forms of mental health researcher and knowledge user partnerships have grown in popularity, primarily in high-income settings, particularly among young people [9]. With most lifelong mental health conditions having an onset by the mid-20s [10], there is a vital focus on better understanding youth mental health needs and curating services tailored to their needs. Several benefits of such youth-researcher collaborations have been identified. For example, coproduction with youth increases the relevance of research aims [11] and facilitation of recruitment [12,13] and helps the production of richer and more reliable data [14]. Researcher-intensive tasks such as data analysis, the presentation of findings, and dissemination also benefit from youth involvement [15]. Coproduction also enables incorporating lived experiences effectively; with regard to user outcomes, coproduction helps young people develop transferable skills [9,16] and research skills [11] and to understand their own mental health better [17].

### Complexities and Challenges of Undertaking Coproduction With Young People

Though coproduction of knowledge may seem like an elegant solution to ensure the implementation of evidence-based practices, it is a highly complex, context-specific experimental process that is often time-consuming and resource intensive [18]. Effective coproduction requires collaborative effort at every stage of the research process [19]. The environment is important, as are researcher traits, such as openness, tolerance, and flexibility [20], and organizational qualities, such as building trustworthy relationships and innovative methodologies [21,22].

The complex process of coproduction with youth comes with specific challenges, as complicated power dynamics may need to be identified, acknowledged, and addressed. Researchers may need to address youth-adult hierarchies [23], which may disrupt effective collaborative work. If such traditional patterns are not disrupted, tokenism, difficulties in conflict resolution and shared decision-making between youth and researchers can ensue [24]. Research processes that ensure coproduction with youth require additional time and resources owing to a number of cultural, ethical, and safety considerations, such as assent-consent procedures for legal minors [25] and ensuring safe practices while also recognizing their agency [5].

### Measuring the Impact of Coproduction

Understanding and evidencing the impact of coproduction with youth remains an area in need of further development. The current literature on the monitoring and evaluation of young people’s involvement in research is diffuse and dispersed and does not have a standard taxonomy or methodology [26]. Moreover, much of the focus has been on “measurable, economic and quantifiable impacts,” which does not take into account the numerous dynamic processes of coproduction [27]. The diversity of approaches, evaluative frameworks, and depth of discourse have made it difficult to measure the impact of coproduction on research processes and outcomes.

Few studies report the impacts of youth coproduction [28], complex processes of collaboration, and nonlinear impacts for stakeholders [29]. The MindKind study used mixed methods to elicit young people’s views on data governance and to assess the feasibility of setting up a user-controlled global mental health databank [30]. This study was conducted in India, South Africa, and the United Kingdom. Participants aged 18 to 24 years (16 to 24 years in the United Kingdom) were enrolled on a separate website and randomized into 1 of the 4 data governance options [30]. Using an app designed for the study, participants were invited to share data on key active ingredients of mental health, such as sleep, body movement, social connections, and positive experiences over a 12-week period. Study participants in the qualitative arm took part in country-specific and multinational group deliberative democracy sessions [31] focused on building a consensus pertaining to the governance of a future mental health databank. Each arm of the study had a strong focus on coproduction.

Given the gaps that exist in the literature, in this study, we aimed to evaluate the impact of coproduction in the MindKind study, and the processes involved in achieving such an impact. We defined the impact of coproduction as a powerful or major influence on 4 stakeholder groups: youth (professional youth advisors and young people’s advisory group [YPAG] members), researchers (staff who contributed to research design, data collection, and analysis), decision makers (principal investigators and funders), and support and administrative staff (broadly defined to include anyone who undertook substantial administrative and support responsibilities as part of their role). We used the impact framework proposed by Beckett et al [27], which applies a multifaceted lens to understand the breadth of coproduction at different levels, the interactions between these levels, and emergent mechanisms. These levels include paradigmatic, structural, organizational, group, and individual levels. Paradigmatic impacts are those impacts of coproduction with the potential to modify ways of understanding and shift frames of reference. Infrastructural impacts include broader social, economic, policy, and political impacts. Organizational impacts include rules, norms (culture), practices, and organizational structures. Group impacts include interpersonal and stakeholder relationships, whereas individual impacts include personal changes such as improved mental or physical health and improved practice and skills. The emphasis within this framework is to “consider longer term developments, wider social changes, any unintended consequences and how coproduced research might affect and be affected by different power dynamics” [27].

We note that the findings discussed in this manuscript do not include any data from the main MindKind study or any of the randomized arms. The objective of this manuscript was to describe the impact of coproduction with youth on different stakeholder groups [32]. Coproduction with youth was designed to influence both the qualitative and quantitative study arms (ie, youth were coresearchers), and we evaluated such impact of youth acting as coresearchers on key stakeholder groups. Advisors in the project did not participate in the qualitative or quantitative study conducted as part of the MindKind study.

## Methods

### Ethics Approval

Ethics approval for the MindKind study was obtained from each of the country sites, namely the United States (WIRB #20212067), the United Kingdom (University of Cambridge, Department of Psychology Research Ethics Committee: Ref. PRE.2021.031 and University of Oxford: Ref R73366/RE00), South Africa (Walter Sisulu University #029/2021 and the Department of Higher Education and Training), and India (India Law Society #ILS/242/2021 and Health Ministry Screening Committee).

### Overview of Youth Coproduction

Each MindKind study site employed a full-time youth lead or professional youth advisor (youth advisor), to convene and run a YPAG in each country site (India, South Africa, and the United Kingdom) and advise on all aspects of the study. The youth advisor was a young person aged 18-23 years who had lived experience of mental health challenges. The in-country YPAGs in each site comprised of young people with lived experience of mental health challenges aged 18-24 years (16-24 years in the United Kingdom), known as YPAG members. In addition to the in-country YPAGs, around 3 to 4 YPAG members from each site joined an international YPAG to advise on broader study questions.

**Figure 1:**
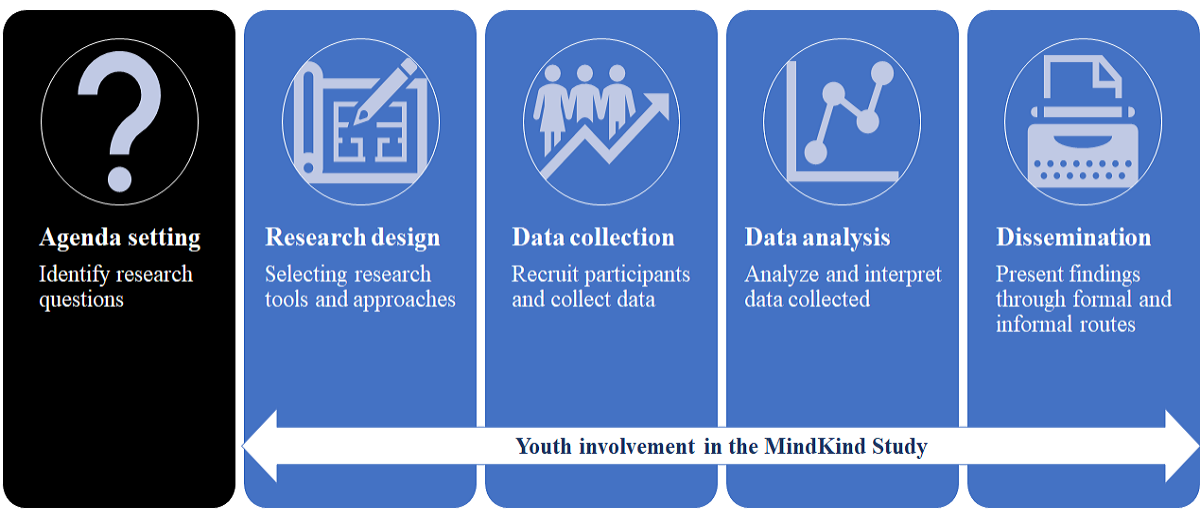
Youth involvement in the MindKind study.

Each youth advisor had 1 vote on the study’s Steering Committee and, therefore, actively participated in study decision-making. Sessions conducted with the in-country and international YPAGs were planned collaboratively by the research teams and led by the respective youth advisors. Sessions were recorded, and feedback shared by the YPAGs was reported by the youth advisors to the research teams via a web-based platform called Airtable. Researchers were then required to respond in Airtable to clarify whether the feedback had been actioned, and if not, why. Youth involvement in MindKind is described in Figure 1.

### Data Sources

Coproduction with youth occurred during the study period between September 2020 and July 2022. Data collection to evaluate the impact of coproduction was conducted sequentially in 2 stages with 4 sets of stakeholder groups: youth (advisors and YPAG members), researchers (staff who contributed to research design, data collection, and analysis), decision makers (principal investigators and funders), and support and administrative staff (defined broadly to include anyone who undertook substantial administrative and support responsibilities as part of their role).

First, we collated various project documents generated between September 2020 and January 2022, such as meeting recordings and minutes, project descriptions, and work plans, to determine the coproduction outcomes relevant to all stakeholder groups (youth, researchers, decision makers, and administrative staff). A total of 17 documents were downloaded from the project database for analysis. A summary of these data sources is presented in Textbox 1. The outcomes identified at this stage allowed us to refine subsequent data collection on significant changes for the 4 stakeholder groups.

Second, building on the identified outcomes, we generated data based on the *Most Significant Change* (MSC) technique on the main changes that resulted from coproduction with youth for the 4 stakeholder groups, that is, moving from the outcomes that were intended to take place to the impacts that actually took place [33]. This technique involved gathering stories of the MSCs from stakeholders via open questions to distill key learning about the study and recommendations for the future. In this study, MSC technique meant gathering views from the 4 stakeholder groups on what had changed or had been impacted as a result of youth coproduction. Researchers and youth advisors implemented the MSC technique via web-based in-depth group discussions with in-country and international YPAGs. In these group discussions with YPAG members, the youth advisor in that study site and the lead authors gathered youth views on what impacts they felt had taken place as a result of youth coproduction.

For other stakeholders (ie, researchers, decision makers, and administrative staff), an open-ended web-based questionnaire solicited views on significant changes across 4 to 5 specified outcomes for each stakeholder group. This web-based questionnaire was emailed to all stakeholders in the study and completed by 16 (52%) out of 31 participants. Although the final sample of respondents was not very large, it involved representation from each of the stakeholder groups and therefore, represented a wide cross section of views. All questionnaire responses were deidentified before analysis.

**Summary of documentary data sources.**

**Initial project discussion notes**

- Youth panel decisions between August and November 2020
- Global Mental Health Databank (initial name of MindKind) feedback documents
- Global Mental Health Databank project structure and governance
- Youth panel engagement
- Youth panel objectives

**Steering committee notes**

- Agenda and meeting minutes
- Presentation on project goals
- Presentation on data usability group feedback and results

**Global youth panel meetings**

- Meeting notes
- Presentation

**Youth advisor onboarding materials**

- Project governance plan
- Proposal for youth involvement
- Youth panel concept note

**Youth advisory planning spreadsheet**

- Youth panel deliverable tracker

**Youth advisory planning presentation**

- Airtable feedback loop

**Funder materials on youth mental health**

- Mental health priority log

### Data Analysis

The analysis focused on building a picture of key changes that occurred for the 4 stakeholder groups as a result of implementing youth coproduction in the MindKind study and understanding how stakeholders perceived its functioning and progress. We used a qualitative approach to collate and synthesize the collected data. Analysis took place at 2 time points: from December to February 2022 and from March to May 2022.

The first stage consisted of a document analysis performed by on of the lead authors (LN). Outcomes aimed to be achieved due to youth coproduction were derived from study documents. These outcomes were then mapped to the impact framework by Beckett et al [27] (Textbox 2). Following this, inputs were gathered from youth advisors, researchers, and YPAG members in each site, and outcomes were revised as a result of such inputs.

The second stage consisted of analyzing the MSC data to identify the MSCs identified by the 4 stakeholder groups that took place as a result of youth coproduction. This analysis was performed collaboratively by 3 researchers, 3 youth advisors, and 7 YPAG members from the United Kingdom and India, who expressed their interest in being involved in data analysis. Web-based sessions with the YPAGs were transcribed, and open-ended survey responses were summarized. These transcripts and summaries were then scrutinized for impacts on the 4 stakeholder groups, which were categorized under the impact framework [27].

In the final stage of analysis, MSC data were coanalyzed by all analysts using an iterative voting approach to increase the reliability of the findings [33]. This involved all analysts discussing the key impacts, identifying the most impactful quotations shared by participants from each of the 4 stakeholder groups, discussing reasons for their choices, and then voting again. A preliminary version of the findings was developed and revised after discussion with advisors and YPAG members.

**Outcomes of coproduction intended to take place, derived from study documents.**

**Outcomes at the paradigmatic level**

- Better conceptualization of study questions, measures, and design
- Reorientating common research practices and assumptions

**Outcomes at the infrastructural level**

- Research that values youth voices
- Exploration of youth preferences and attitudes on a range of topics pertinent to data governance and mental health
- Youth-informed dissemination strategies
- Empowerment of young people as data users

**Outcomes at the organizational level**

- Established communication channels between all stakeholders
- Accountability mechanisms for receipt of feedback
- Increased connection between youth and research institutions
- Differentiated information outputs based on different information needs of stakeholders
- Time and effort invested in team building to fully integrate youth

**Outcomes at the group level**

- Engaging and authentic relationships between stakeholders, especially between youth and other stakeholders
- Youth-driven rules of engagement for advisors and young people advisory group members

**Outcomes at the individual level**

- Increased knowledge and capacity of research concepts and processes (for youth) and contexts of youth lives (for other stakeholder groups)
- Adequate compensation for time and effort; networking and skill development opportunities; insights into own mental health experiences; feeling heard (for youth)

## Results

### Overview

A total of 31 stakeholders working across all MindKind study sites, belonging to the 4 stakeholder groups, participated in group discussions and questionnaires: 11 youths (3 advisors; 7 members across YPAGs in India, South Africa, the United Kingdom and an international YPAG; and 1 lived experience consultant at the funding organization); 11 researchers in India, South Africa, the United Kingdom, and the United States; 6 decision makers (individuals responsible for key study decisions, such as principal investigators and youth); and 3 support and administrative staff. All participants were employed in the MindKind study and had <1 to 25 years of health research experience. Where stakeholders belonged to more than 1 group, the primary group affiliation was used for the analysis (eg, a professional youth advisor could belong to the youth, researcher, and decision maker stakeholder groups). The participants were based in both high- and middle-income settings.

We found that impact occurred at 5 levels detailed in the framework developed by Beckett et al [27], namely, the paradigmatic, infrastructural, organizational, group, and individual levels. Detailed findings are available in Multimedia Appendix 1.

### Paradigmatic Impact: Big-Picture Learnings for Coproduction

Paradigmatic impacts are those impacts of coproduction with the potential to modify ways of understanding and shift frames of reference [27]. We identified 2 paradigmatic impacts of coproduction in the MindKind study: first, coproduction enabled new and unexpected ways of conducting research, and second, coproduction modified research priorities and influenced study conceptualization and design, although this was not always successfully implemented.

With regard to the first impact of new ways of conducting research, we observed that youth advisors prompted researchers and decision makers to engage with diverse groups of youth to advise as part of YPAGs and use innovative methods to recruit study participants (eg, using Instagram stories and posts to reach young people). For YPAGs, advisors ensured that recruited members represented diverse backgrounds in research experience, ethnicity, geographic location, gender, or disability status. Previous research shows that YPAG recruitment is limited in diversity and geographic reach (eg, more advisors who are women with prior research experience, based locally) [11]. However, improved digital and technological flexibility in web-based coproduction enabled greater diversity.

Key impacts of youth coproduction were the introduction of Airtable, weekly youth-focused meetings, and relationship building among individual site teams. These immediate impacts then helped improve the process of coproduction by creating separate spaces for youth to share feedback, enabling practices for all stakeholders to engage with one another, and setting up multiple avenues for examining how coproduction goals were being met. Accountability mechanisms are also important for tracking the implementation of generated ideas:

We have to create spaces to explicitly solicit young people’s feedback; just “having them in the room” may be too overwhelming an environment to engage.

Researcher

Second, coproduction with youth transformed research priorities and activities and influenced study conceptualization and design. Through coproduction, we identified points of dissonance between different stakeholder groups and used them to improve study activities, key research decisions, and research knowledge generated to align more with youth priorities, life experiences, and contexts. One example of this was to introduce more capacity building activities for advisors and YPAGs as a priority to effectively advise and participate in research implementation. Without such capacity building activities, youth stakeholders can find it difficult to provide timely and effective advice, and researchers and decision makers find it challenging to incorporate youth perspectives in research. A shared mission and common goals were important for guiding the overall direction of coproduction.

YPAG members and advisors advised on the structure and content of the MindKind app, such as preferred app engagement features, which were a core component of the quantitative study. They also reviewed and shared feedback on questions designed to be used in qualitative group discussions with the study participants. However, some of their feedback on the MindKind app could not be successfully incorporated into the app development process because of the research and funding timelines. Timing was critical, as a number of early decisions had to be made (owing to logistical and funding constraints) before the coproduction systems had been set up. This highlights the importance of timing and early engagement; otherwise, there was a risk of disengagement among youth stakeholders:

If one is going to be doing research around youth, then involve youth from the beginning right through to the end. I think MindKind didn’t quite do this as we had already developed the App and the areas to focus on before we started the youth advisory groups...we did a bit of “roughshodding” and hoped they would agree with us!

Decision maker

Capacity building activities for youth to advise on research implementation and dissemination needed significant additional time and resources, especially from advisors and early career researchers, which were exacerbated by restricted funding timelines. These constraints meant that there were contexts in which coproduction was limited, and the study had to consider meaningful ways of course correction or acknowledge which parts of the study could not be meaningfully coproduced. However, systems for reflection in multiple teams, forums, and different groups were critical in ensuring that meaningful engagement was always possible, and attempted, even if not successful in every instance:

Many discussions around involvement have revolved around equality. However, I thought it was important to emphasise equity. This meant taking a unique approach which focused on youth capacity development and ongoing reflexivity rather than assuming involvement just meant a seat at the table.

Youth stakeholder

### Infrastructural Impact: Constraints and Opportunities in Existing Systems

We describe infrastructural impacts of coproduction on dissemination activities, as well as infrastructural constraints within research, administrative, and funding structures that governed the larger MindKind study.

Coproduction had positive impacts on research dissemination for both formal (the MindKind final report, journal publications, and conference presentations) and informal (blog posts, seminar discussions, and internal presentations) outputs. For example, we coanalyzed and cowrote our youth coproduction publications with advisors and YPAG members, which introduced views and perspectives that might not have otherwise been incorporated. Advisors at each site (India, South Africa, and the United Kingdom) shared their views on the topics to be addressed in publications arising from the study. They also contributed to preparing an outline for each publication, participated in documentary analysis, and shared key learnings from youth involvement via blog posts. Advisors and YPAG members also coanalyzed qualitative data by assisting with theme and subtheme selection, voting on the most important findings to be highlighted, and commenting on drafts of the manuscript.

We were regularly invited to share our learnings with external research groups who had either begun or were contemplating undertaking coproduction in the form of informal discussions or extended presentations. We presented the challenges and opportunities of coproduction in posters at academic conferences, internal seminars, and university meetings (eg, within the Child and Adolescent Psychiatry research group at the Department of Psychiatry, University of Oxford). One such presentation at an internal seminar was led entirely by the advisor and YPAG members. The presence of a full-time advisor was a positive catalyst for learning in other research projects.

Factors that facilitated positive infrastructural impact included greater funding and administrative support to ensure that these tensions were addressed appropriately. Flexible timelines and the timely involvement of youth were also critical in ensuring that youth stakeholders could be fully integrated and included in the project as equal partners in research. This also meant that research funding was critical to timing, as coproduction needed resources available at the funding application stage to be done appropriately.

Infrastructural impacts varied across contexts. For example, the youth advisor and YPAG members in South Africa frequently faced planned electricity outages or “load shedding,” which hampered their ability to meet via web-based portals on a regular basis. YPAG members in South Africa also did not consistently have access to the internet, and their agreed remuneration was delayed owing to wider issues with the financial administration, both of which negatively impacted their ability to engage. In contrast, advisors in India and the United Kingdom faced fewer infrastructural issues, which aided regular engagement and advice from YPAG members. These findings underscore the importance of infrastructure in a wider sense, including access to the internet and responsive administrative systems that were essential for facilitating effective coproduction.

An additional infrastructural impact within this study was that we experienced a constant tension between project timelines, deliverables, and coproduction aims. To undertake coproduction with youth fully, we needed to invest significant time and effort on capacity building activities, tailoring existing systems, and mentorship, but these were not always compatible with the study timelines. Pressure to produce reports or updates in line with project milestones were also often at odds with the time that was needed to effectively coproduce a specific aspect of the study, whether that was about the design, data collection, or analysis.

### Organizational Impact: Differentiated Communication Practices and the Feedback Loop

Organizational impacts include impacts on rules, norms (culture), practices, and organizational structures [27]. We found that the most critical organizational impact involved changes in communication. Coproduction prompted organizations to engage appropriately with diverse stakeholder groups, including (1) ensuring that a feedback loop was in place to communicate with stakeholders, especially youth, and that this loop was closed in a way that stakeholders felt heard and (2) implementing differentiated communication practices for different stakeholders.

With regard to the feedback loop, we set up an Airtable system to record feedback from YPAG members and advisors. Research and project teams then acted on the feedback. If implementing feedback was not possible, they explained why it could not be done. Although this was an encouraging start, we found that the critical component was ensuring that feedback loops needed to be closed in a way that the youth felt they had been addressed. Data collection on Airtable also had to be made more conversational to engage with youth, so formal data collection platforms were supplemented by meetings where youth could share their feedback verbally, in addition to written feedback:

Communication must be done continuously and through multiple channels. Because everyone learns and contributes differently.

Decision maker

Given that effective communication meant something different for each stakeholder group, our communication practices needed different frequencies and levels of detail as well as a more inclusive information-sharing strategy to include youth. This sometimes meant that given the large number of stakeholders to consult, decisions needed more time to be taken. We implemented other changes to address these issues, for example, a weekly digest email for important announcements; quick links to project documentation; and a list of the upcoming week’s meetings, including any scheduled YPAGs. Importantly, the digest was designed to ensure that everyone, including the advisors, was informed about the decisions to be made, who was making them, and when and in what format feedback would be welcomed. These digests became important tools for increasing transparency and inclusivity, directly impacting communication between stakeholders.

### Group Impact and Interpersonal Relationships: Authenticity and Cycles of Engagement

Group-level impacts constitute interpersonal and stakeholder relationships within a system [27]. We observed that due to coproduction, authentic relationships developed between researchers and advisors and between advisors and YPAG members, and YPAG members experienced cycles of engagement and disengagement.

Authentic relationships among the advisors were aided by creating multiple channels of communication with them and among them. The advisors and study team met every week during the course of the study to discuss updates, youth integration, challenges, and learnings. The youth advisors also met each other, together with an external lived experience consultant and advisor (employed by the funder), for regular check-in meetings. They also had a monthly scheduled check-in with each other.

Advisors were responsible for leading their own YPAG and were encouraged to foster authentic relationships with the members. They met the YPAG on a fortnightly basis and conducted individual check-ins with the members to understand any concerns or feedback from them. Overall, the project team treating the advisors and YPAG members with curiosity and professionalism was helpful in fostering these relationships.

Providing the YPAG members with opportunities to upskill and spending time and resources on engagement were important in creating positive group relationships. For example, actively soliciting youth feedback, trying innovative ideas, and offering multiple avenues for youth to engage and contribute to the study beyond the role of an advisor (involvement in capacity building activities, manuscript ideation, and writing) helped increase engagement. Furthermore, closing the feedback loop in a way felt by the youth was helpful, but when this was not possible, it could lead to disengagement if not properly communicated by researchers and decision makers:

Some key things to consider when engaging and retaining youth in the MindKind Study—Providing due credit to young people where necessary and allowing them to be key decision makers who hold equal power as adult staff on the team; Providing compensation/reimbursement/honorarium for the time that youth engage with us; Providing young people with constant opportunities to upskill (through workshops, manuscript involvement, consultancies on projects aside from MindKind); Ensuring that young people are given support when needed (emotional/support with work etc.)

Youth

Collaboration between researchers and advisors was established over time and was beneficial to overall youth engagement. This was done through multiple channels, such as organizing check-ins between researchers and advisors, as well as directly involving advisors in all research-related activities.

### Individual Impact: Better Skills, Knowledge, and Capacities

According to Beckett et al [27], individual impact constitutes the characteristics of stakeholders, including biological and psychological aspects such as improved mental or physical health and improved practice and skills for practitioners. For advisors and YPAG members, major individual impacts included greater insight into one’s own mental health; increased knowledge of research concepts and processes; opportunities to harness lived experiences of mental health challenges; remuneration for time and skills; and new academic, presentation, and study skills. The advisors and YPAG members reported developing better insights into their own mental health by participating in project activities and having discussions about a future global mental health databank. Furthermore, they reported increased skills and knowledge development in research-related concepts:

One of the most significant changes for me is probably the importance of research and studies which, which I mean, are now involved in but just in general, for the understanding of mental health...it’s helped me look at mental health as more collective thing, rather than being more focused on the individual just because, like we, we convene, we talk on a weekly basis, but also the moving parts and the systems which have to be in place for some sort of change to happen. And some sort of, like, insight into mental health and research to actually come to fruition.

Youth

These impacts were mediated by the following factors: having site-specific and international YPAGs led by advisors, capacity building activities for advisors to independently coordinate and lead several study components, regular capacity building activities for YPAG members to advise on study, and dedicated spaces for youth to interact with each other. These capacity building activities involved dedicating sessions to demonstrate research in practice. Customized support for advisors to suit their backgrounds and interests was also critical for enhancing outcomes:

Young people’s social and cultural contexts affect their knowledge and opinions on mental health. While this was something that I had some idea about prior to the study, having regular conversations with advisors and researchers across the sites led to interesting learnings on how social and cultural contexts play a role in shaping a young person’s perspective and knowledge on mental health (for example, what mental health means to a young person in a HIC [high-income country] vs what it means to a young person in an LMIC [low- and middle-income country]).

Youth

For researchers and decision makers, enhanced coproduction with youth resulted in a much richer understanding of young people’s life experiences and contexts:

I think we learned a lot about how youth wanted to be engaged...the decision making on the side of the research team needed to happen at a much faster pace than the youth were comfortable (or able) to make decisions. As a result, we sometimes just “told” rather than “asked.” I think the youth forced us to slow down and demanded inclusion, which was a very powerful change to the study.

Decision maker

This was aided by ongoing reflexivity by the research team and decision makers as well as course correction based on youth feedback.

## Discussion

### Summary of Findings

Our findings suggest that coproduction with youth in the MindKind study had significant positive impacts for all stakeholder groups (youth, researchers, decision makers, and administrative and support staff). In addition, having a web-based YPAG allowed engagement from a diverse group of panel members, facilitating wider reach and convenience by conducting sessions at suitable times. We found that coproduction impacted, to varying degrees, all 5 domains of practice: paradigms (new or innovative ways of doing research and influence on research priorities, conceptualization, and design), infrastructure (improved dissemination of learning and constant tensions between study timelines and coproduction owing to infrastructural stressors), organizational (differentiated communication needs, practices, and outputs; greater emphasis on closing the feedback loop; and better access between youth and research institutions), group (more authentic relationships among youth and between youth and other stakeholders, and ebbs and flows in engagement), and individual (increased skills and capacities for all, opportunity to use lived experience of mental health for youth, and increased knowledge of youth life experiences for researchers and decision makers). Some impacts, such as societal or infrastructural impacts, occurring beyond the project can be challenging to measure and document but cannot be ignored.

### Implications and Recommendations

Youth coproduction generally led to better outcomes overall, not only for youth but also for researchers, decision makers, administrative staff, and the research as a whole. Individual- and group-level impacts included increased knowledge, skills, and capacities for youth and other stakeholders; youth being able to use their lived experience of mental health and feeling heard; authentic relationships between the YPAG and advisors; and ebbs and flows in engagement over the project life cycle. Many of these findings align with the wider literature on the impact of coproduction with youth [4,28,34]. Having a dedicated youth advisor in each site was critical in achieving these impacts. There was no full-time youth advisor in the US study team (ie, not a study site, but the coordination hub); if present, this might have resulted in improved communication and greater youth input into some of the technological decisions.

Coproduction with youth consistently challenged nonyouth stakeholders on their assumptions of what the research could look like. This aligns with the wider literature, which emphasizes that the involvement of stakeholders in research is linked with greater effectiveness, if there is articulation of a shared mission and goals [35]. Our findings further confirm that although a shared mission is critical, establishing clear expectations with funders and organizations in terms of the practical costs of undertaking coproduction and implementing necessary changes in organizational communication practices are critical in achieving better outcomes for all.

Our experience of coproduction with youth as an active process of valuing all types of knowledge and experiences and creating a balance between different stakeholders aligns with others’ experiences [36]. However, we encountered several barriers in implementing coproduction with youth according to its tenets and in line with our shared mission [37]. Our systems for reflection and discussion were critical in addressing these barriers effectively, which is a characteristic of effective partnerships [35]. Our findings point to the need for ongoing monitoring and evaluation of impact and to ensure that this is set up as early as practicable in the research cycle. Such monitoring and evaluation should take the form of first articulating outcomes and measurable indicators of what coproduction is meant to achieve, for whom, and by when. Establishing feedback forms and recurring surveys that capture periodic progress toward these goals is a good starting point.

Coproduction was enacted, implemented, and received differently by stakeholders in India, South Africa, and the United Kingdom (MindKind study sites). Equally, public and institutional infrastructure (eg, access to the internet) also played a significant role in the way coproduction was implemented and received in different contexts. Although such challenges broadly map to the literature on coproduction in low- and middle-income countries versus high-income countries [28], we found variations between South Africa and India in this context. Our findings underscore the concept that coproduction is highly “place based” and occurs in particular social, economic, and ecological contexts [38].

An important organizational impact detected was the change in communication practices and outputs needed to undertake coproduced research and ensuring that there was a feedback loop in place and stakeholders (especially youth) felt that this was closed in an appropriate way. This begins with the recruitment and integration of youth advisors, understanding their varied communication needs, and changing communication and decision-making practices. These findings are consistent with previous literature that highlights that effective coproduction is achieved when there are positive working relationships within and between teams, stakeholders feel heard, and tailored communication helps create shared meanings of concepts [35]. Our key mechanisms of achieving this impact included establishing feedback loops and testing them to ensure that they were fit for practice and supporting administrative staff to implement these communication practices. These practices are characterized as “maintenance tasks” in the wider literature that supports the functioning of partnerships by addressing core administrative and support needs [35].

The relatively recent completion of the study limits attempts to describe the wider infrastructural or societal impact as intended in the framework by Beckett et al [27]. Instead, we discuss modest impacts on dissemination and infrastructural constraints while undertaking coproduction with youth. However, to determine the wider infrastructural impact of youth involvement at the societal level, coproduction with youth would need to be assessed at the national scale, where such methods are adopted to measure its public value. Our experiences broadly map the practical costs and challenges of coproduction, such as large administrative burdens, increased researcher time and resources, the lack of training on the implementation of coproduction, and insufficient funds and resources to effectively undertake coproduced research [39]. Although we found significant benefits of undertaking coproduction, we also found that grappling with timelines and costs could lead to a diluted version of coproduction to *coproduction lite*. Despite the focus and funding support, our experience highlights the entrenched structural challenges in undertaking coproduced scientific research more broadly [40]. Our findings highlight the need to cocreate the infrastructure needed to undertake coproduction, resource it appropriately, and recognize the time and skills needed to coproduce research [39].

### Youth Involvement

We were committed to ensuring the meaningful involvement of young people, but funding and timeline constraints meant that such involvement needed to be proportional and pragmatic. Therefore, we involved YPAG members in areas where they had particular knowledge, interests, or skills, specifically data collection, analysis, and the interpretation of findings. We analyzed the data in collaboration with advisors and YPAG members who were interested in doing so and jointly interpreted the findings and decided on specific findings to be presented in the paper. In relation to dissemination, we also met YPAG members and advisors who had participated in data analysis and interpretation to understand how they would like to be acknowledged in the paper (in this study, as coauthors).

### Conclusions

Although the resources involved in coproduction are significant, they yield several benefits for various stakeholder groups and across several domains. We have attempted to capture these impacts and their enabling or disabling factors in further detail in the context of the MindKind study, which focused on young people’s views of mental health data governance in India, South Africa, and the United Kingdom. We found that “effective coproduction emerges in practice” [41], and numerous opportunities and challenges arise in the implementation of coproduction.

There are no one-size-fits-all coproduction efforts. In sharing the impact of coproduction on various stakeholders and outcomes, we hope to have additionally demonstrated the value of measuring and reporting outcomes associated with youth coproduction [28]. We recommend that monitoring, evaluation, and learning systems be designed and implemented early in coproduction studies to enable more systematic reporting of coproduction with youth and its impacts. Future research should also examine how we can standardize the reporting of youth involvement in research; the Guidance for Reporting Involvement of Patients and the Public (GRIPP) checklist provide a useful starting point [42]. More frequent and detailed reporting of the impact of coproduction with youth will likely challenge the current funding and research infrastructure constraints and enable better health research outputs.

## Appendix

Multimedia Appendix 1 Impact, processes, and mechanisms of coproduction with youth in the MindKind study.

## Abbreviations

GRIPP: Guidance for Reporting Involvement of Patients and the Public
MSC: Most Significant Change
YPAG: young people’s advisory group
