# Supplementary material for: Evidencing the Impact of Web-Based Coproduction With Youth on Mental Health Research: Qualitative Findings From the MindKind Study
Source: JMIR Public Health Surveill. 2023 Jun 19;9:e42963. doi: 10.2196/42963 (PMC10365598; doi:10.2196/42963)
Supplement: Multimedia Appendix 1 [file publichealth_v9i1e42963_app1.docx]

| Impact level | Research process | Impacts | Key mechanisms | Select quotations |
| --- | --- | --- | --- | --- |
| Paradigmatic | Adhering to the principles of coproduction | *New and innovative ways of conducting research:*   - Developing innovative methods to engage and retain youth panel members in diverse contexts - Incorporating accountability mechanisms into research - Acknowledging that coproduction is messy but creating spaces to learn and reflect - Promoting digital/technological flexibility in research   *Modified research priorities, study conceptualization and design:*   - Understanding dissonance or points of divergence between youth and other stakeholders - Incorporating capacity building initiatives in research - Incorporating youth priorities (may/may not include research) in study design but this is not always successful | - Good or bad timing can greatly impact outcomes, so coproduction must be timed appropriately - Costs and benefits of coproduction are both high, so finding the right balance between barriers and facilitators is key - Coproduction is messy, and there are times we will fall short, so making space to reflect and learn is critical - Shared mission and goals that are clearly articulated | - “We need timely involvement and for this to happen in this project it would have not been feasible as the delays to the project would have been too great and so we need to be mindful that we do not make the barriers to coproduction so high that no research gets done, so finding ways to make this feasible for other projects- as not all projects will have recourse to the funding but might be impactful. It is also quite a substantial investment and probably better to have YPAGs^a^ that are shared across projects to make them more sustainable.” [Decision maker] - “It is important to build a trustworthy and meaningful relationship with the youth when soliciting feedback from them. Young people must be provided with opportunities that will provide them with an advantage or incentive. Engaging them on only one aspect/part of the study will lead to a dip in engagement. Use simple and easy language when communicating with the youth. Avoid research jargon. Understand a young person’s context and how it affects their communication and participation in research. Ensure that enough support is provided to them to meaningfully participate in discussions (emotional support, capacity building for research etc.)” [Youth] - “My monthly spaces with advisers were a chance to debrief, reflect and connect with one another as well as share knowledge and learnings. These spaces were a brilliant opportunity to link involvement across sites, especially in a project with many meetings and high levels of complexity.” [Lived experience adviser] |
| Infrastructure | *Infrastructural actors involved and how:*  Higher education institutions in study sites and United States; nonprofit organizations in South Africa and United States; charities in the United Kingdom | - Project timelines and coproduction aims: constant tensions between the two - Knowledge outputs (presentations, journal articles, and blog posts) coproduced with youth - Greater administrative support to ensure adherence to coproduction principles - Coproduction highly dependent on context | - Administrative support that is resourced appropriately - Tension between project demands (funding and timelines) and coproduction is a reality; planning for this and constantly reviewing this is key - Timely involvement of youth is critical in designing an inclusive system - Addressing infrastructure access in specific contexts (eg, access to electricity and internet) | - “The project was designed to move fast—faster than a study usually would. But meaningfully involving youth is done by creating time and space for those engagements. There was a constant tension between meeting deadlines and involving youth meaningfully.” [Decision maker] - “Supporting meaningful youth involvement is time consuming, and therefore costly. Significant time and effort must be expended on capacity building, contextualizing, and mentorship if youth will be expected to contribute honest and actionable feedback as members of the team. It is not possible to meaningfully incorporate youth leadership into a project by simply including the youth in the project meetings.” [Support staff] |
| Organizations | *Institutions involved and how:*  Higher education institutions in study sites and United States; nonprofit organizations in South Africa and United States; charities in the United Kingdom | - Increased emphasis on closing the feedback loop and ensuring that youth feedback is addressed (or reasons provided for why it could not be addressed) - Increased bridging of gaps between youth and research institutions - Differentiated communication practices to engage varied stakeholders | - Establishing processes for closing feedback loops - Understanding communication preferences of different stakeholders - Supporting administrative staff to create customized reporting and communication structures | - “The major learning for me is the importance of ensuring youth feel that the feedback loop has been closed. We did a poor job of this early on in the project and I think our AirTable forms, as well as the weekly digest emails from [Staff] and the structure of our Monday meetings helped in closing the feedback loop and ensuring things did not fall through the cracks. But this is still a work in progress!” [Researcher] - “By virtue of the fact that we were brought on board as panelists, I think, the way we gave inputs, it was almost immediately inculcated or noted down. And in the next meeting we would see the suggestions being incorporated. I definitely felt like I was a part of the research group. Even when we were making decisions about the study, I think we were making informed decisions, since we were presented with certain existing data to take a call before sharing any suggestions. We shared from our personal experiences too, but along with data informed decisions.” [Youth] |
| Group(s)/interpersonal relations | *Interpersonal relationships:*  Relationships between the following groups: advisers and wider site teams and coordinating institutions in the United States; YPAG members and advisers; researchers across study sites and coordinating institutions in the United States; support and administrative staff with all others | - Authentic relationships between advisers and panel members - YPAG members feeling engaged and invested, and at other times, feeling disengaged - Long-term relationships and collaborations between researchers and advisers | - Treating advisers and YPAG members with curiosity and professionalism - Opportunities for youth to upskill - Spending time and resources on engagement (eg, actively soliciting youth feedback, trying innovative ideas, and offering multiple avenues for youth to engage) - Providing non-youth staff with resources and time to build relationships | - “I think researchers could benefit from being digitally/technologically flexible. Platforms like WhatsApp, Miro, Zoom chat, etc. have provided rich opportunities for exchange, and especially in an online-based study, ‘traditional’ researchers need to adapt to the tools that work.” [Researcher] |
| Individual | *People involved:*  YPAG members in India, South Africa, and the United Kingdom and international YPAGs and advisers in India, South Africa, and the United Kingdom; researchers in India, South Africa, the United Kingdom, and the United States; decision makers in India, South Africa, the United Kingdom, and United States; and support and administrative staff in India, South Africa, the United Kingdom, and the United States | - Greater insight into own mental health (youth) - Increased knowledge of research concepts and processes (youth) - Opportunities to harness lived experience of mental health challenges (youth) - Developed new academic, presentation, and study (youth) and knowledge translations skills (researchers, support staff, and decision makers) - Increased knowledge of young people’s life experiences and contexts (researcher and decision makers) | - Site-specific and international YPAGs led by advisers - Capacity building activities for advisers to independently coordinate and lead several study components - Capacity building activities for YPAG members to advise on study - Dedicated spaces for youth to interact with each other - Customized support for adviser to suit their backgrounds and interests - Ongoing reflexivity | - “It has been really interesting to see the level of knowledge that young people have about data use, which reinforces the need to provide a solid education about these things. For example, a lot of young people involved in research were not aware that data is generally pseudonymized and made very difficult to identify individuals. I have also become a lot more aware of barriers faced by young people in low- and middle-income countries—including lack of internet access, load shedding, and difficulties accessing public facilities because of safety concerns.” [Researcher] - “It has really helped with school, sometimes learning things during the panel. Having an external knowledge source.” [Youth] - “I think we learned a lot about how youth wanted to be engaged...the decision making on the side of the research team needed to happen at a much faster pace than the youth were comfortable (or able) to make decisions. As a result, we sometimes just ‘told’ rather than ‘asked’. I think the youth forced us to slow down and demanded inclusion, which was a very powerful change to the study.” [Decision maker] |
